# Vaccinating against COVID-19: The Correlation between Pro-Vaccination Attitudes and the Belief That Our Peers Want to Get Vaccinated

**DOI:** 10.3390/vaccines9111366

**Published:** 2021-11-20

**Authors:** Darie Cristea, Dragoș-Georgian Ilie, Claudia Constantinescu, Valeriu Fîrțală

**Affiliations:** Faculty of Sociology and Social Work, University of Bucharest, 010181 Bucharest, Romania; dragos.ilie@unibuc.ro (D.-G.I.); claudia.constantinescu@unibuc.ro (C.C.); valeriu.firtala@unibuc.ro (V.F.)

**Keywords:** vaccines, sociological survey, social norms, vaccine acceptance, vaccine hesitancy, COVID-19 vaccination, pro-vaccination attitude, pandemic, society

## Abstract

This study verifies whether there is a strong correlation between the pro-vaccination, against COVID-19 attitude of the respondents and their belief that most of those around them want to be vaccinated against COVID-19. For this purpose, we analyzed data from a sociological survey conducted in April 2021 in Romania. The sample size was of 1001 respondents, the selection process was randomized and the population included in the sample is representative of the socio-demographic structure of Romania. The tool used to collect the data was CATI (telephonic interview). In order to test the existence of these correlations we performed the following tests: Chi-Square test, Kendall τ, Spearman ρ tests and Freeman’s z-test. The pro-vaccination attitude strongly correlates with the perception of subjects that their primary group accepts vaccination and even correlates with the perception that the general public is rather pro-vaccination. The vaccination decision is closely linked to the social relations system and the rules of the community in which the subject lives. In this paper we discuss the correlation between attitude and belief, not the existence of a causal relation between the two of them.

## 1. Introduction

We tested for the existence of a correlation between the acceptance of vaccination against COVID-19 by the general population and the belief that other people accept vaccination. The study is based on a nationally representative opinion poll for the population of Romania in which we actively participated as researchers. In order to achieve this, we introduced in the survey a question regarding the subject’s intention to vaccinate, a question regarding the belief that most of their acquaintances are in favor of vaccination, and also a question regarding the belief that most Romanians are in favor of vaccination.

From the data of a 2019 ‘Eurobarometer’, we can observe that the majority of Romanians, more than half, tends to have a positive attitude towards vaccination [1]. In a second European Commission study on the attitude of Europeans towards vaccination against COVID-19, which was published in June of 2021, only 57% of Romanians said that they would vaccinate as quickly as possible; this result placed Romania in 21st place out of 27 European Union member countries in terms of the population’s desire to vaccinate as quickly as possible [2]. Even if 57% of Romanians say they will get vaccinated as soon as possible, official data show the opposite, i.e., only 24.9% (as per August 2021) of the population has been fully immunized by vaccination, meaning only 4,834,109 people got vaccinated [3].

An analysis of COVID-19 vaccine acceptance published in the journal Nature was performed across 15 survey samples in Asia, Africa, South America, Russia and the United States. The authors used data collected in 2018 on acceptance of vaccination [4] and coverage rates of childhood vaccines [5] to understand the “prospects for COVID-19 vaccine uptake”. The results appeared to be hopeful for the acceptance of COVID-19 vaccines. However, the conclusion of the study shows multiple variations in the attitude and acceptance “across and within countries, including in settings with high acceptance of other vaccinations” [6].

The conclusions of the report delivered in October 2020 by World Health Organization (WHO)’s technical advisory group on behavioral insights and sciences for health advanced three strategies to be adopted for the identified drivers of vaccine uptake: creating an enabling environment, harnessing social influences and increasing motivation [7]. In April 2021, a survey conducted in United States before the COVID-19 vaccine was developed was published [8]. The results describe as independent variables “perceptions of risk, exposure to different media and platforms for COVID-19 news, political party identification, trust in science, and social determinants of health including education, income and race and ethnicity”. The findings of a recent study published in June 2021 show that the likelihood of individuals to take the vaccine or to vaccinate their children increases when they are surrounded by positive attitudes or their desire is to comply with family, friends/peers behavior [9].

We do not intend to make an extensive review of the explanations in the literature on the public’s appetite for vaccination. This has been done many times during this difficult period which, unfortunately, has relaunched the vaccination controversy. Note, however, that openness to anti-COVID-19 vaccination is not necessarily similar to agreement with vaccination in general, as discussed before the pandemic that began in 2020 [10,11].

There are several sociological models that could explain the population’s appetite for vaccination. Synthetically, most fall into two large groups: models centered on public trust in the government and in the medical system, and models centered on public confidence in science and vaccines (opposite to a type of traditionalism, trust in parallel medicine or, more complicatedly, trust in conspiracy theories). The first refers rather to what we call trust in vaccination, and the second rather to what we call trust in the vaccine. These are two slightly different phenomena, depending on what you focus on, the process or the substance. These two groups of explanations are important and can work together but, from our research, we want to draw attention to a third paradigm. That can explain the decision to vaccinate or not, and give very good direction on conducting communication campaigns to popularize the vaccine, especially in situations where this conduct must be promoted quickly and massively.

The explanatory model we have identified is a purely psychosociological one, of public opinion. It is based on the perception of a person, who is faced with the decision to vaccinate or not, on the informal social norm in his or her group of close friends. Of course, even in this case, several factors with psychosociological relevance can be taken into ac-count. However, our focus is on how the perception of vaccination as normal correlates with the acceptance of vaccination, such an approach being even easier to use in the construction of a probationary campaign. Our idea is based on a theoretical psychosociological approach which is quite well known. We refer to studies on the influence of social norms, attitudes, and perceptions on people’s intention to address a particular type of behaviour. In practice, our adoption of certain types of behaviors is influenced by a whole range of attitudes and perceptions of how we perceive social (informal) norms, social practices of those “like us”, the expectations that we believe that those in our narrower or broader group have in relation to us and that range of behaviors [12,13].

The perspective was successfully applied in a report conducted in Romania by the Romanian Foundation Angel Appeal for UNESCO on the adoption of risk behaviors by adolescents [14]. However, since the quoted research concerned risk behaviors (smoking, alcohol and drug use, certain sexual behaviors), it was rather aimed at behaviors more or less in the sphere of deviance. If we think that accepting a new vaccine, in the midst of a medical crisis, is a behavior that, at least at first, can be perceived as a carrier of risks, but in no way is it located in the area of deviance or illicit, comparing our intention to adopt this behavior with what we think other people, closer or farther away, are doing, it’s all the more likely.

We must also mention an experimental study published in 2019 in The Lancet [15] which describes the complex way in which social norms influence the intention of subjects to receive the influenza vaccination. The research does not study the link between the perceived group norm and vaccination, but only between the interest/intention of vaccination and exposure to pro-vaccination messages that suggest that a certain percentage of the population in that area has been vaccinated. The conclusion of the article is that, if the mobilizing messages suggest that a very large percentage of the local population is vaccinated, the interest in vaccination decreases because the perception of danger disappears. If the messages communicate an average incidence of vaccination in the population from within the area, vaccination becomes attractive. If the indicated vaccination coverage is low, the interest is also low, because the subject does not perceive a social norm in this respect.

There are two situations here: the reference to public opinion in general (and the reference to the subject’s peers and family. We operate here with a simple but classic distinction in sociology, that between society (the first situation) and community (the second situation) [16].

We studied the association between the vaccination intention and the two mentioned items using the following association tests: Chi-Square, Spearman’s ρ, Kendall’s τ and Fisher’s “z-test”. Based on our test, we identified that there is a very strong correlation between accepting vaccination and the belief that acquaintances of the respondents get vaccinated. We also identified a strong correlation between accepting vaccination and the belief that other Romanians are getting vaccinated.

Our hypothesis is that the perception of social norms as being pro-vaccination is associated with the subject’s pro-vaccination attitude. We are talking about the perception of the informal social norm–what people do and think about the vaccine.

## 2. Materials and Methods

The data on the basis of which we conducted this study were collected in a larger opinion poll, considered nationally representative for Romania [17]. The study was conducted under the auspices of the Institute of Political Science and International Relations of the Romanian Academy of LARICS, a public opinion analysis laboratory well known in Romania. The data were gathered between 12 April and 23 April 2021 using a random, probabilistic and multi-layered sample. The methodology used was quantitative. The sample volume was 1001 persons and is representative of the noninstitutionalized population of Romania, aged 18 years and above. The method used was a survey based on a computer-assisted telephone interview (CATI). The interviews were applied to the resident population in Romania, in all residence environments, including the capital of Bucharest. The sample was validated on the basis of official public data of the National Statistical Institute of Romania [18].

This study looked at the existence of a correlation between the individual’s attitude towards vaccination against the SARS-COV2 virus and the individual’s perception of what his acquaintances have towards the subject. The three variables were measured ordinally. For the variable that measured the individual attitude towards vaccination, the subjects had the possibility to declare themselves in favor of vaccination, already vaccinated, reserved or against, the order being from positive to negative [19]. For the variable that measured how the individual perceives the attitude of his acquaintances towards vaccination, we asked if most of his acquaintances wanted to vaccinate, the response options being “True/False”, and the same method of measurement was also used for the variable that encompassed the individual’s perception of the attitude of other Romanians towards vaccination.

Our first null hypothesis was “There is no association between the attitude towards the vaccine and the opinion of the acquaintances”, and the alternative hypothesis was “Individuals who have a positive attitude towards vaccination think about their acquaintances that they want to vaccinate”.

The second null hypothesis is “Attitude towards vaccination is not correlated with the attitude of the entire population about vaccination”, and the alternative hypothesis is “Individuals who have a positive attitude towards vaccination think about other Romanians that they want to vaccinate”.

## 3. Results

We performed a chi-square test on the variable ‘Attitude towards COVID-19 vaccination’ with the variables ‘Most Romanians rather want to be vaccinated against COVID-19’ and ‘Most of my acquaintances want to be vaccinated against COVID 19’. The results of these tests can be observed in Table 1.

As it can be observed from Table 1, both *p*-values are below 0.005. This tells us that there is a statistically significant relationship between the respondent’s attitudes towards vaccination against COVID-19 and how to they perceive that their acquaintances and conationals feel about vaccination against COVID-19. This result tells us that people who are pro-vaccination (against COVID-19) tend to perceive others as being pro-vaccination and skeptics tend to perceive others also as skeptics.

We wanted to test the strength of the association between the variables after establishing that there is a statistically significant correlation between them. In order to do so we decided to apply further testing in the form of the “Kendall τ (tau-b)” test, the “Spearman ρ (rho)” and the ‘z-test’.

As we measure the correlation between two ordinal variables, we decided to use the “Kendall τ (tau-b)” test and the “Spearman ρ (rho)” coefficient test, where if the resulting score is close to zero, this indicates a lack of association, and a value between −1 and 1 shows a strong association between the two variables [20]. For data analysis we used the statistical processing software SPSS IBM Statistics.

We decided to recode the answers to the question “Do you vaccinate yourself against COVID-19?”, from four variants of the answer, namely “Yes; I’ve already been vaccinated; No. I certainly don’t want to get vaccinated; I’m still thinking about getting vaccinated in the next period; Non-answers”, in a new variable that had only two values “Pro-Vaccination”, which included the answers “Yes; I’ve already been vaccinated”, and “Skeptics”, that included the rest of the answers. We decided to treat non-responses as part of the skeptical group. We consider that a lack of an answer in such a context and to a fairly clear question is rather a masking of the respondents’ skepticism.

Table 2 shows that almost 60% of the sample is skeptical about the intention to vaccinate against C.

From Table 3 one can observe a roughly equal distribution between the two variants of response, which is slightly over 50%, considering that their acquaintances intend to vaccinate against COVID-19.

Table 4 shows that 52% of respondents believed that the majority of Romanians would like to vaccinate against COVID-19.

The first test we performed was to cross-examine the two variables: the individual’s attitude towards vaccination with the perception of the attitude of the acquaintances towards vaccination. The result can be seen in the chart below.

**Figure 1:**
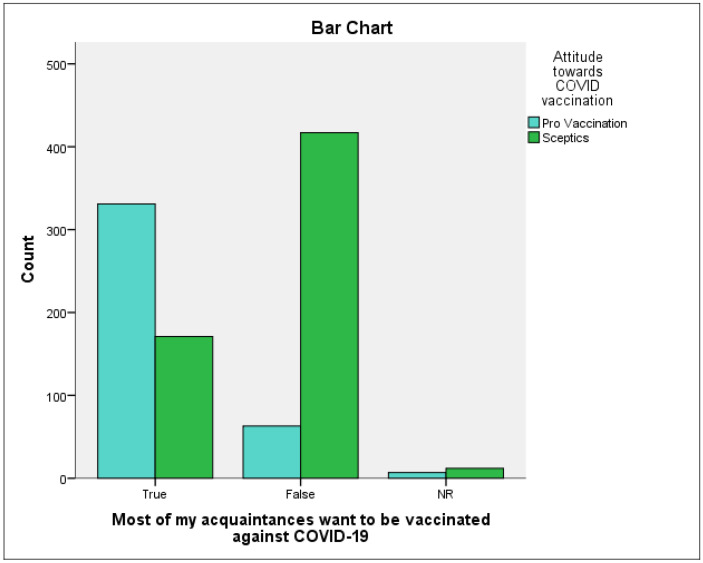
The individual’s attitude towards vaccination vs. the perception of the acquaintances’ attitude towards vaccination.

From the graph below (Figure 1) we can see quite clearly a grouping of individuals around their attitudes towards vaccination and how they perceive the attitude of their acquaintances. Those who have a pro-vaccination attitude tend to believe that their acquaintances (friends, family, etc.) have a similar attitude towards vaccination.

To ensure the strength of this association we decided to perform two tests: the test of “Kendall τ” and the test of the coefficient “Spearman ρ”. The results can be seen in Table 5.

By following the Sig coefficient (*p*-Value), we can observe a score of 0.000, which is less than 0.05 and which shows us that the null hypothesis is invalid [21]. Analysing the Spearman ρ (rho) correlation test, it scored 0.516, which shows a strong correlation between the two variables. Kendall τ coefficient’s score is 0.512, which also indicates strong association between the two variables [19].

By comparing based on Fisher’s “z-test” the differences between the values of the average scores of the two populations we obtained tare shown in Table 6:

Looking at Table 6 above, we can identify that there are significant differences between those who are skeptical about the vaccine and those who are pro-vaccination in terms of the variable that measures the attitude of the acquaintances towards vaccination.

We may say, with a fairly high degree of confidence, that there is a strong association between the two variables, and that the phenomenon of vaccination is causally linked with the way the individual perceives his social environment. We may also say that there are statistically important differences that arise between the two populations. So the null hypothesis is not validated; therefore the hypothesis “Individuals who have a positive attitude towards vaccination think about their acquaintances that they want to be vaccinated” is a valid one.

To verify the second null hypothesis we carried out the same tests that we applied to the previous hypothesis, and the results of the test can be observed in Table 7.

**Figure 2:**
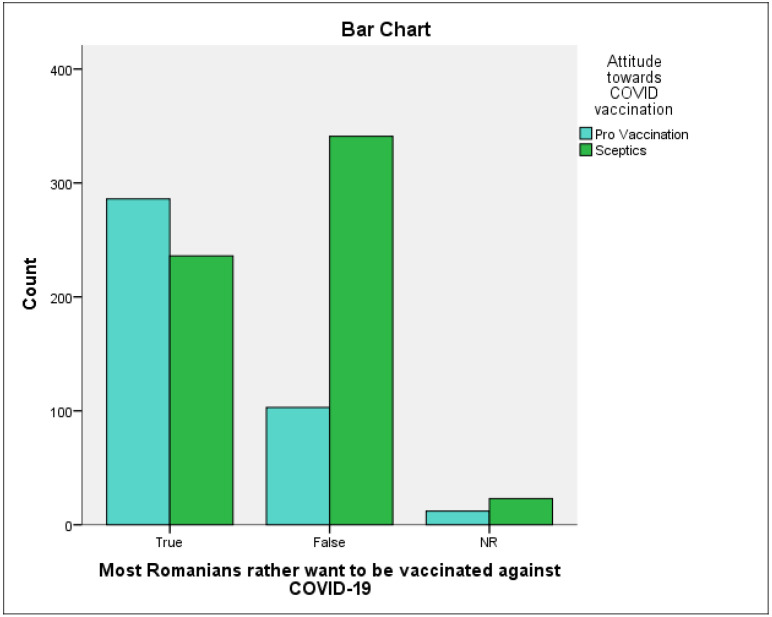
Individual’s attitude towards vaccination vs. the perception of the attitude of fellow countrymen towards vaccination.

From the graph (Figure 2) below we can see the existence of an association between the two variables: the attitude of the individual towards vaccination and the perception of the attitude of his co-nationals towards vaccination, even if it seems to be weaker than in the case of his acquaintances.

After repeating the two tests we obtained a significance coefficient (*p*-Value) of 0.000, identical to that of the variable in the previous hypothesis, a Kendall’s τ score of 0.297653 and a Spearman’s ρ score of 0.302301. *p*-value is sufficient to invalidate the null hypothesis, and the Spearman’s ρ score of 0.30 indicates the existence of an average positive association, but which is weaker than in the case of the first hypothesis, in which we had a Spearman ρ score of 0.516.

In the case of the ‘z-test’, as can be seen in Table 8, applied to these two variables, it was found, as in the case of the first hypothesis, that there are significant differences between the two groups, namely those who have a pro-vaccination attitude and those who are skeptical. Pro-vaccination individuals tend to declare that the rest of their co-nationals also have a pro-vaccination attitude, while skeptics tend to believe the opposite.

As you can see from Table 9, 82.5% of subjects who are in favour of COVID-19 vaccination believe that their acquaintances are also in favour of vaccination. The percentage is obviously significant, but let’s look at the other statistical tests.

## 4. Discussion

In the analysis below we will talk strictly about the data on vaccination against COVID-19. We therefore carried out the correlation tests and the results show that there is a positive association between the intention to vaccinate and the belief that the acquaintances also will be vaccinated. There is also a correlation between the pro-vaccination attitude and the belief that Romanians want to get vaccinated, although this association is much weaker than the first. There is certainly a correlation between the intention to vaccinate/pro-vaccination attitude and the belief that the acquaintances and the people/Romanians in general are favourable to vaccination. The perceived regulatory pressure of the small, community group, is more relevant than the perception of a general social norm.

There are some studies that have researched the correlation between socioeconomic factors and attitudes toward vaccination against COVID-19 [22,23], some that talk about differences in vaccine intake between populations around the globe [24], and one that talks about the vaccine intake among different gender, age and other sociodemographic factors in Austria [25]. These papers focus on the general differences between social groups and not on the relation between the individual and her/his peers. Our paper tries to identify a relation between the way the individual perceives the social norm in their group and his or her actions. Even if we do not argue that there is a causality between the decision to get vaccinated and the way an individual perceives others around him or her are getting vaccinated, we cannot ignore the strong correlation between the two.

We argue that our findings show that the decision to get the vaccination or to refuse vaccination has also a social dimension. This dimension is normative and is influenced by the way in which individuals perceive social norms, as discussed in the Introduction of this paper [11,12,13]. Understanding that vaccination can be a social fact or a social action that is correlated with the individual’s perception of social norms may help policy makers in their efforts of stopping the pandemic or, by extrapolation, in making future public policies more efficient. The discussion that is emerging around this research is not necessarily why Romania’s population is reluctant to vaccinate, but rather around the social contexts in which the individual lives and how they come to influence their decisions. The association between the attitude towards vaccination against COVID-19 of the individual and that of his acquaintances indicates that vaccination has a social character; we do not choose to vaccinate ourselves to protect those around us, we rather choose to vaccinate ourselves so as not to be marginalized in our group of acquaintances. If the individual’s group of acquaintances considers vaccination against COVID-19 important for whatever reason, then we might expect the group members to either limit their interactions with the unvaccinated or try to persuade the unvaccinated to vaccinate.

That is why the attitude of individuals towards vaccination is closely related to how they come to understand the attitude of their countrymen or that of their close friends and family in relation to this problem.

### 4.1. Limits of Our Research

Even though this study was carried out at a national level and the level of representativeness was calculated at the level of the Romanian population, since it is a CATI data collection, we cannot exclude the limitations brought about by this instrument. The fact that the questions in this article were part of a larger study on various topics, which had an average response time of 20 min, may constitute a limitation of the research, since respondents cannot be asked more questions to deepen the understanding of the problem of vaccination against COVID-19. We consider that a study dedicated exclusively to identifying the causes that lead to the associations identified by us would be necessary. Another limitation is the level of measurement used for the variables in our hypotheses, a measurement using a ten-step scale could have shown us the degrees of confidence or reluctance that respondents have in relation to vaccination against COVID-19.

### 4.2. Possible Further Developments of the Paper

As we explained, our goal was to highlight the existence of a correlation between the pro-vaccination attitude and the perception that others will be vaccinated/are pro-vaccination. We did not intend to discuss whether there is a causal relationship behind this correlation. We intend to take such an approach in a later article that will be based on data that we currently collect (we already have another field survey underway) and on their statistical processing suitable for demonstrating causality (regression, factor analysis, etc.).

## 5. Conclusions

Our data show a very strong link between the favourable attitude towards vaccination and the perception that our acquaintances and even people in general also have a positive attitude towards vaccination, in the case of vaccination against COVID-19.

If there is a causal relationship between the perception of others as also favourable to vaccination and one’s own pro-vaccination attitude, this is something that can be discussed further. However, it was not the goal of our paper to discuss that. We only intended to highlight the strong correlation between the two.

The correlation we have identified is certain and has a pragmatic utility in defining pro vaccination campaigns: if subjects see that the social norm of their group or of those like them is an attitude of pro-vaccination, it is very likely that this contributes strongly to the acceptance of vaccination.

The question may arise as to why this is important. In general, people do what other people do. Uncertainty prevailed at the beginning of the vaccination campaign against COVID-19. The question was if the world would accept the new vaccine. Back then, the public did not have a clear benchmark of the social norm. In April, the month in which our data was collected, vaccination against COVID-19 in Romania was still in its infancy and it was unclear whether the social norm would be vaccination or vaccine refusal.

People often tend to form their own perception of what is normal or not, especially in situations of uncertainty. In our case, coherence seems to have increased in the face of uncertainty: whoever wants to be vaccinated is convinced that those around him/her also want to be vaccinated. Who does not want to be vaccinated is convinced that most others also will not. Whoever is convinced that vaccination is the social norm, wants to be vaccinated. Whoever is not convinced of that fact, does not want to be vaccinated.

## Figures and Tables

**Table 1:** Results for the chi-square tests.

Pearson Chi-Square	Value	df	Asymp. Sig. (2-Sided)
Most Romanians rather want to be vaccinated against COVID-19 vs. Attitude towards COVID-19 vaccination	100.223	2	0.000
Most of my acquaintances want to be vaccinated against COVID-19 vs. Attitude towards COVID-19 vaccination	285.093	2	0.000

**Table 2:** The respondents’ attitude towards COVID-19 vaccination.

Variables	Frequency	Frequency	Percent	Valid Percent	Cumulative Percent
Valid	Skeptics	600	59.9	59.9	59.9
	Pro-vaccination	401	40.1	40.1	100.0
	Total	1001	100.0	100.0	

**Table 3:** The respondents’ perception towards COVID-19 vaccination of their acquaintances.

Response	Frequency	Percent	Valid Percent	Cumulative Percent
Non-Response	19	1.9	1.9	1.9
My acquaintances are not getting vaccinated against COVID	480	48.0	48.0	49.9
My acquaintances are getting vaccinated against COVID	502	50.1	50.1	100.0
Total	1001	100.0	100.0	

**Table 4:** The respondents’ perception towards COVID-19 vaccination of their fellow countrymen.

Response	Frequency	Percent	Valid Percent	Cumulative Percent
Non-Response	35	3.5	3.5	3.5
My countrymen are not getting vaccinated against COVID	444	44.4	44.4	47.9
My countrymen are getting vaccinated against COVID	522	52.1	52.1	100.0
Total	1001	100.0	100.0	

**Table 5:** Results of Kendall τ and Spearman ρ tests.

Tests	Correlations	Attitude towards COVID-19 Vaccination	Most of My Acquaintances Want to Be Vaccinated against COVID-19
Kendall’s τ			
Attitude towards COVID-19 vaccination	Correlation Coefficient	1.000	0.512 **
	Sig. (2-tailed)		0.000
	N	1001	1001
Most of my acquaintances want to be vaccinated against COVID-19	Correlation Coefficient	0.512 **	1.000
	Sig. (2-tailed)	0.000	
	N	1001	1001
Spearman’s ρ			
Attitude towards COVID-19 vaccination	Correlation Coefficient	1.000	0.516 **
	Sig. (2-tailed)		0.000
	N	1001	1001
Most of my acquaintances want to be vaccinated against COVID-19	Correlation Coefficient	0.516 **	1.000
	Sig. (2-tailed)	0.000	
	N	1001	1001

** Correlation is significant at the 0.01 level (2-tailed).

**Table 6:** Provaccination vs. skeptical population on ‘Most of my acquaintances want to be vaccinated against COVID-19’.

Question	Response	Attitude towards COVID-19 Vaccination	
		Pro Vaccination (A)	Sceptics (B)
**Most of my acquaintances want to be vaccinated against COVID-19**	True	B	
	False		A
	NR		

**Table 7:** Kendall τ and Spearman ρ results for hypothesis 2.

Tests	Correlations	Attitude towards COVID-19 Vaccination	Most Romanians Rather Want to Be Vaccinated against COVID-19
Kendall’s τ			
Attitude towards COVID-19 vaccination	Correlation Coefficient	1.000	0.298 **
	Sig. (2-tailed)		0.000
	N	1001	1001
Most Romanians rather want to be vaccinated against COVID-19	Correlation Coefficient	0.298 **	1.000
	Sig. (2-tailed)	0.000	
	N	1001	1001
Spearman’s ρ			
Attitude towards COVID-19 vaccination	Correlation Coefficient	1.000	0.302 **
	Sig. (2-tailed)		0.000
	N	1001	1001
Most Romanians rather want to be vaccinated against COVID-19	Correlation Coefficient	0.302 **	1.000
	Sig. (2-tailed)	0.000	
	N	1001	1001

** Correlation is significant at the 0.01 level (2-tailed).

**Table 8:** Pro-vaccination vs. skeptical population on ‘Most Romanians rather want to be vaccinated against COVID-19’.

Question	Response	Attitude towards COVID-19 Vaccination	
		Pro-Vaccination (A)	Sceptics (B)
Most Romanians rather want to be vaccinated against COVID-19	True False NR	B	A

**Table 9:** Attitude towards COVID vaccination vs. Most of my acquaintances want to be vaccinated against COVID-19.

Attitude towards COVID Vaccination		Most of My Acquaintances Want to Be Vaccinated against COVID-19			Total
		True	False	NR	
Sceptics	Count	171	417	12	600
	% within Attitude towards COVID vaccination	28.5%	69.5%	2.0%	100.0%
Pro Vaccination	Count	331	63	7	401
	% within Attitude towards COVID vaccination	82.5%	15.7%	1.7%	100.0%
Total	Count	502	480	19	1001
	% within Attitude towards COVID vaccination	50.1%	48.0%	1.9%	100.0%

## Data Availability

The results of the survey can be publicly viewed at this link: https://larics.ro/wp-content/uploads/2021/06/Barometru-de-Securitate-a-Romaniei_CCSL_ISPRI_aprilie-2021.pdf (accessed on 20 October 2021).
